# Prevalence and risk factors of congenital heart defects among live births: a population-based cross-sectional survey in Shaanxi province, Northwestern China

**DOI:** 10.1186/s12887-017-0784-1

**Published:** 2017-01-13

**Authors:** Leilei Pei, Yijun Kang, Yaling Zhao, Hong Yan

**Affiliations:** Department of Epidemiology and Health Statistics, School of Public Health, Xi’an Jiaotong University Health Science Center, Xi’an, Shaanxi 710061 People’s Republic of China

**Keywords:** Congenital heart defects, Epidemiologic characteristics, Northwestern China

## Abstract

**Background:**

Nearly half of the population of Northwest China live in Shaanxi province, but population-based data on the epidemiologic characteristics of congenital heart defects (CHD) in this population is limited. The study aimed to investigate the prevalence and epidemiologic characteristics of the CHD among infants born between 2010 and 2013 in Shaanxi province.

**Methods:**

Infants born between 2010 and 2013 in Shaanxi province were surveyed using a stratified multi-stage sampling method. Participant characteristics were recorded by questionnaire, medical records were reviewed and CHD was diagnosed using a specialized neonatal echocardiography. A Poisson regression model was applied to assess the association between any CHD and possible risk factors.

**Results:**

A total of 29098 live infants were surveyed with an overall prevalence of 76.0 (95% CI: 66.3, 86.7) per 10000 live infants. The prevalence of major and minor CHD were 26.1 and 49.8 per 10000 live infants, respectively, in surveyed areas. Poisson regression analysis indicated that, compared with singleton infants, the prevalence rate ratio of CHD was higher in twin and multi-fetal infants (PRR:3.1, 95% CI:1.6, 6.1). Using southern Shaanxi as a reference, the PRR of CHD were lower in northern (PRR:0.4, 95% CI:0.3, 0.6) and central Shaanxi province (PRR:0.5, 95% CI:0.4, 0.7). PRR was higher in mothers over 30 years of age than in those under 25 years (PRR:1.6, 95% CI:1.0, 2.5), and in mothers with ≥3 parity than that in mothers with only one parity (PRR:2.2, 95% CI:1.2, 4.2). The risk for CHD among live infants was positively associated with family history of CHD (PRR: 9.8, 95% CI: 5.3, 18.1). Additionally, CHD was less common in the floating population than the permanent population (PRR: 0.6, 95% CI: 0.4, 0.9).

**Conclusion:**

The CHD among live infants seemed to be a serious health problem in Shaanxi province as well as in Northwestern China. Our research have important policy implications for recommendations on CHD intervention in Northwest China.

**Electronic supplementary material:**

The online version of this article (doi:10.1186/s12887-017-0784-1) contains supplementary material, which is available to authorized users.

## Background

Congenital heart defects (CHD) are one of the most prevalent and serious birth defects, and are the leading causes of birth defects-associated morbidity, mortality, and medical expenditures [1–3]. Many studies in developed Western countries have estimated that approximately 40 to 100 per 10000 live births suffer from CHD, and 26 to 44 per 10000 births from major defects, defined as defects requiring catheter or surgical intervention during the first year of life [1, 4–7]. As complex congenital heart defects can cause spontaneous abortion or stillbirth, the reported incidence of CHD among fetuses (146.0 per 10000 fetuses) may be underestimated [8]. In China, CHD is the most prevalent birth defect, accounting for over 10% of cases of infant mortality in 2008 [9, 10], and surgical treatment of infants with CHD is reported to cost 12 billion Yuan each year in China [11].

In Sparsely populated Northwestern China, economic condition are worse and health services are less developed than in Eastern and Coastal areas of China. Nearly half the population of this region, 37 million people, reside in Shaanxi province. In a population-based cross-sectional survey of the whole province conducted in 2013, we have found that the prevalence of birth defects among alive infants is high in Shaanxi province of Northwest China and cardiovascular system malformations are the most common [12]. Until recently, however, limited population-based data on the main epidemiologic characteristics of CHD in Shaanxi province or Northwest China were available. The objective of the study was to investigate the prevalence and epidemiologic characteristics of CHD among live infants born between 2010 and 2013 in Shaanxi province. We expect the results to provide the most updated information on the rate of CHD among infants in Shaanxi province, Northwest China.

## Methods

### Study design and participants

A large population-based cross-sectional survey was conducted between August and November 2013 in Shaanxi province, northwest China. Infants born between 2010 and 2013, who were alive at the time of sampling, were evaluated in the study. Considering the imbalanced population distribution between rural and urban areas in the province, a stratified multi-stage sampling method was employed. In rural areas, counties, townships and villages are administered individually, while in urban areas the three administrative districts are districts, streets and communities. Twenty counties in rural areas were selected randomly throughout the province. In each county, 6 townships were chosen and 6 villages from each chosen township were sampled randomly. To account for differences in population density and fertility level between rural and urban areas, 10 districts in urban areas were randomly selected, 3 streets in the chosen districts and 6 communities in the chosen streets, were selected randomly.

Finally, approximately 30 mothers and their babies in each chosen village and 60 mothers and their babies in each chosen community were selected using a completely random sampling method. According to the 2010 sixth census, the population of Shaanxi was approximately 37 million, and the fertility rate was 9.7‰ [13]. A total of 29098 live infants were eventually enrolled in the study, representing a relative sampling size of nearly 9.00% of the total infant population.

### Data collection

In the survey, all questionnaires including birth defects questionnaire, family questionnaire, reproductive history questionnaire and family history questionnaire were designed by Xi’an Jiaotong University Health Science Center (Additional file 1: Questionnaire). A pilot study was carried out to pretest all questionnaires and procedures, and detailed interviewer guides were developed. Field surveys and data collection were carried out by the trained field staff from Xi’an Jiaotong University Health Science Center. Ten investigation teams from Xi’an Jiaotong University Health Science Center were established for these counties or districts. Each team consisted of 10 to 12 investigators, a supervisor and a doctor from a local maternal and child health hospital. During the survey, all fieldworkers were closely monitored by their supervisors and randomly examined. When errors and/or missing values were detected, participants were re-interviewed immediately. All the information was collected in the local village clinics and community health service centers. Meanwhile, our work was supported by the local hospitals and health administrative departments and the Ministry of Health in Shaanxi province.

After this study was fully explained to participants, the informed consent was obtained. A face-to-face interview was conducted to collect socioeconomic and demographic characteristics of mothers and their children using the family questionnaires. A multistage process determined the presence of a congenital heart defect in each child. First, the infants were screened for CHD by a doctor from the local maternal and child health hospital. Isolated patent foramen ovale and patent ductus arteriosus among neonates <28 days of life were excluded because they are normal neonatal findings.

For those affected by CHD, an in person interview was conducted with their mothers and medical records reviews were reviewed by a trained physician. Parents were also required to recall the CHD diagnosis process and the time of diagnosis, diagnosing hospital, type of CHD were recorded from medical records. For the new cases of suspected CHD, a specialized neonatal echocardiography and electrocardiography was conducted at the first affiliated hospital of Xi’an Jiaotong University Health Science Center, and participants were diagnosed accordingly.

### Main study variables

To present an accurate picture of the burden of CHD, we classified CHD as major or minor. Defects were classified as major when the gross structural complexity was of functional significance, e.g. hypoplastic left heart syndrome (HLHS), atrioventricular septal defect (AVSD), coarctation of the aorta (CoA), and large ventricular septal defects (VSD), large atrial septal defect (ASD), and surgical repair or catheter intervention was likely to be required. The most common major heart defects (e.g. AVSD, VSD, HLHS, ASD) were further grouped as simple or complex. A simple heart defect was defined as one without additional cardiac defects, e.g. AVSD or HLHS, while a complex heart defect was one with additional heart defects, e.g. HLHS with additional coarctation of the aorta. Defects were classified as minor when no intervention was likely to be required, e.g. mild ventricular septal defects (VSD), mild atrial septal defect (ASD), mild pulmonary valve stenosis (PS). Due to less impacts of the defects on infants’ life and the privacy of infants, some parents of infants with minor CHD refused to have their infants further checked. Therefore, the type of some minor CHD did not be determined and they were classified as unspecified CHD.

The socioeconomic status of the households was assessed using the Demographic and Health Survey wealth index [14]. Principal component analysis was used to combine the variables representing families’ economic level. On the basis of the first principal component, then, the socioeconomic status of the families was classified into the tertiles representing poor, medium and rich families. According to the residence during the pregnancy, women were categorized into two groups (permanent or floating). The floating population included women who lived in surveyed areas for less than three months during pregnancy. Permanent residents were those who lived in surveyed areas for more than three months during pregnancy. Other characteristics of the participants were also entered into the multivariate analysis, including number of ultrasound examination, infant gender (male or female), fetal number (singleton or twin and multi-fetal), family history of CHD among first-degree relatives (yes or no), registered permanent residence (urban or rural), mother's education (no education, primary, junior high, senior high, or college and above), mother's age (<25, 25–29 or ≥30 years groups), parity (1, 2 or ≥3), and areas (north, central or south).

### Statistical analysis

Data was entered into Epidata 3.1 by double entry (CDC, Atlanta, GA, USA) and all statistical analysis was performed using STATA version 12.0 software (STATA Corporation, College Station, TX, USA). Baseline characteristics were compared across different areas using analysis of variance (ANOVA) and *χ* ^2^ test. Prevalence rate (per 10000 live births) and its 95% confidence intervals (95% CI) of CHD based on Poisson distribution across different areas in the province were also presented. Any congenital heart defects were considered unique outcome variables in the multivariate analysis. The Poisson regression model was applied to assess the association between the outcome variable and covariates, including number of ultrasound examination, infant gender (male as a reference or female), fetal number (singleton as a reference or twin and multi-fetal), family history of CHD among first-degree relatives (no as a reference or yes), registered permanent residence (urban as a reference or rural), residence during the pregnancy (permanent as a reference or floating), mother's education (no education, primary, junior high, senior high, and college and above as a reference), mother's age (<25 as a reference, 25–29 and ≥ 30 years groups), parity (1 as a reference, 2 and ≥ 3), and areas (north, central and south as a reference). A prevalence rate ratio (PRR) was quoted as the output from Poisson regression and could indicate the magnitude of change, which would not be negative and <1 for a decrease and >1 for an increase.

## Results

### Baseline characteristics of the participants

A total of 1605 villages were sampled within 272 townships in Shaanxi province and 29098 live infants were studied. The average age of children was 16.9 ± 11.3 months (range 0–46 months). The baseline characteristics of the participants across different areas in Shaanxi province were summarized in Table 1. The proportions of the participants from north, central and south of Shaanxi, were 25.7, 53.9 and 20.3% respectively. More than half of participants resided in rural areas, and 11.3% were characterized as floating.

**Table 1:** Baseline characteristics of study population by different areas in Shaanxi province ^a^

Baseline characteristics	Northern Shaanxi *n* (%)	Central Shaanxi *n* (%)	Southern Shaanxi *n* (%)
Number of ultrasound examination^b^	2.54 (0.8)	2.63 (0.7)	2.58 (0.9)
Infant Gender^b^			
Male	4222 (56.4)	8303 (53.5)	3189 (54.0)
Female	3263 (43.6)	7295 (46.5)	2722 (46.0)
Fetal number			
Singleton	7396 (98.7)	15522 (98.8)	5662 (98.9)
Twin and multi-fetal	100 (1.3)	184 (1.2)	65 (1.1)
Mother's education^b^			
College and above	1044 (13.9)	3860 (24.6)	398 (6.7)
Senior high school	1156 (15.4)	3616 (23.0)	1045 (17.7)
Junior high school	3649 (48.7)	7242 (46.2)	3541 (60.0)
Primary school	1307 (17.4)	839 (5.3)	821 (13.9)
No education	336 (4.5)	133 (0.8)	92 (1.6)
Mother's age, year^b^			
< 25	2209 (29.8)	3227 (20.9)	1792 (30.8)
25–29	3313 (44.6)	6866 (44.5)	2148 (36.9)
≥ 30	1899 (25.6)	5348 (34.6)	1874 (32.2)
Parity^b^			
1	3770 (50.6)	9514 (60.8)	3423 (58.4)
2	3291 (44.2)	5673 (36.2)	2292 (39.1)
≥ 3	393 (5.3)	463 (3.0)	150 (2.6)
Wealth index^b^			
Poor	2740 (36.5)	5199 (33.1)	1706 (28.8)
Medium	2322 (31.0)	5461 (34.8)	1890 (31.9)
Rich	2435 (32.5)	5048 (32.1)	2331 (39.3)
Family history of CHD^b^			
No	7403 (99.2)	15559 (99.4)	5883 (99.7)
Yes	59 (0.8)	92 (0.6)	19 (0.3)
Residence during the pregnancy^b^			
Permanent	6913 (93.2)	13640 (87.2)	5120 (86.7)
Floating	502 (6.8)	1996 (12.8)	785 (13.3)
Household registration^b^			
Urban	1632 (21.8)	3957 (25.2)	423 (7.1)
Rural	5865 (78.2)	11751 (74.8)	5504 (92.9)
Total	7497 (25.7)	15708 (53.9)	5927 (20.3)

^a^Values are given as mean (SD) or the number (percentage) of the study population by socio-demographic characteristics across different areas

^b^denotes significantly differences in characteristics of study population across different areas by *χ*2 test or analysis of variance

Of the participants, 98.8% were singletons and boys accounted for 54.4%. It should be noted that approximately 0.6% of participants had a family history of CHD among first-degree relatives. Participants were evenly divided between poor, medium and rich households. The mean age of mothers was 28.0 ± 4.9 years and the proportion of women who had attended senior high school and above was close to 38.2%. Amongst the mothers, the average of the parity was 1.7 ± 0.8. Enrolled mothers underwent approximately 2.6 prenatal ultrasound examinations during pregnancy. Almost all the baseline characteristics of the study population differed between the three areas in Shaanxi province.

### The prevalence of congenital heart defects

Among the live infants surveyed, we identified 221 cases of CHD, and an overall prevalence of 76.0 (95% CI: 66.3, 86.7) per 10000 infants. To clarify the category of CHD among live infants in Shaanxi province, CHD were subcategorized as major (76 cases) and minor (145 cases). The most common categories of major CHD were large atrial septal defect (ASD; 8.9 per 10000 [95% CI: 5.8, 13.1]), large ventricular septal defect (VSD; 6.9 per 10000 [95% CI: 4.2, 10.6]) and atrioventricular septal defect (AVSD; 4.1 per 10000 [95% CI: 2.1, 7.2]). Moreover, there were 5 cases with tetralogy of Fallot (ToF) and 5 cases with pulmonary valve stenosis (PS). Of the minor CHD, there were 47 unspecified congenital heart diseases. The prevalence of any CHD in the south of Shaanxi province was significantly higher than in north or central Shaanxi province (Tab. 2).

**Table 2:** Prevalence and categories of CHD among infants by different areas in the Shaanxi province^a^

Defects^b^	Northern Shaanxi		Central Shaanxi		Southern Shaanxi		Total	
	*n*	per 10000 (95% CI)	*n*	per 10000 (95% CI)	*n*	per 10000 (95% CI)	*n*	per 10000 (95% CI)
Major CHD	18	24.1 (14.3, 38.0)	36	22.9 (16.1, 31.7)	22	37.2 (23.3, 56.3)	76	26.1 (20.6, 32.7)
ASD,total	6	8.0 (2.9, 17.5)	15	9.6 (5.3, 15.8)	5	8.4 (2.7, 19.7)	26	8.9 (5.8, 13.1)
ASD,simple	3	4.0 (0.8, 11.7)	5	3.2 (1.0, 7.4)	3	5.1 (1.0, 14.8)	11	3.8 (1.9, 6.8)
ASD,complex	3	4.0 (0.8, 11.7)	10	6.4 (3.1, 11.7)	2	3.4 (0.4, 12.2)	15	5.2 (2.9, 8.5)
VSD,total	5	6.7 (2.2, 15.6)	11	7.0 (3.5, 12.5)	4	6.8 (1.8, 17.3)	20	6.9 (4.2, 10.6)
VSD,simple	3	4.0 (0.8, 11.7)	6	3.8 (1.4, 8.3)	2	3.4 (0.4, 12.2)	11	3.8 (1.9, 6.8)
VSD,complex	2	2.7 (0.3, 9.7)	5	3.2 (1.0, 7.4)	2	3.4 (0.4, 12.2)	9	3.1 (1.4, 5.9)
AVSD,total	2	2.7 (0.3, 9.7)	5	3.2 (1.0, 7.4)	5	8.4 (2.7, 19.7)	12	4.1 (2.1, 7.2)
AVSD,simple	2	2.7 (0.3, 9.7)	5	3.2 (1.0, 7.4)	4	6.8 (1.8, 17.3)	11	3.8 (1.9, 6.8)
AVSD,complex	0	0.0 (0.0, 4.9)	0	0.0 (0.0, 2.3)	1	1.7 (0.1, 9.4)	1	0.3 (0.1, 1.9)
ToF	1	1.3 (0.1, 7.4)	2	1.3 (0.2, 4.6)	2	3.4 (0.4, 12.2)	5	1.7 (0.6, 4.0)
PS	3	4.0 (0.8, 11.7)	0	0.0 (0.0, 2.3)	2	3.4 (0.4, 12.2)	5	1.7 (0.6, 4.0)
HLHS	1	2.7 (0.1, 7.4)	2	0.6 (0.2, 4.6)	1	1.7 (0.1, 9.4)	4	1.4 (0.4, 3.5)
CoA	0	0.0 (0.0, 4.9)	1	0.3 (0.1, 3.5)	1	1.7 (0.1, 9.4)	2	0.7 (0.2, 2.5)
EA	0	0.0 (0.0, 4.9)	0	0.0 (0.0, 2.3)	2	3.4 (0.4, 12.2)	2	0.7 (0.2, 2.5)
Minor CHD	19	25.4 (15.3, 39.7)	83	52.9 (42.1, 65.5)	43	72.6 (52.6, 97.9)	145	49.8 (42.1, 58.6)
Mild ASD	8	10.7 (4.6, 21.1)	31	19.7 (13.4, 28.0)	17	28.7 (16.7, 46.0)	56	19.2 (14.5, 25.0)
Mild VSD	5	6.7 (1.6, 11.7)	19	12.1 (7.3, 18.9)	8	13.5 (5.8, 26.6)	32	11.0 (7.5, 15.5)
Mild PS	1	1.3 (0.0, 4.9)	7	4.5 (1.8, 9.2)	2	3.4 (0.4, 12.2)	10	3.4 (1.6, 6.3)
Unspecified CHD	5	6.7 (2.2, 15.6)	26	16.6 (10.8, 24.3)	16	27.0 (15.5, 43.9)	47	15.8 (11.9, 21.5)
Any CHD	37	49.5 (34.8, 68.2)	119	75.8 (62.8, 90.7)	65	109.8 (84.8, 140.0)	221	76.0 (66.3, 86.7)

^a^Values are the number and proportion (95% CI) of CHD in the study population across different areas

^b^ *Abbreviation*: *AVSD*, atrioventricular septal defect, *ASD* atrial septal defect, *VSD* Ventricular septal defect, *HLHS* hypoplastic left heart syndrome, *CoA* Coarctation of the aorta, *ToF* Tetralogy of Fallot, *PS* Pulmonary valve stenosis, *EA* Ebstein’s anomaly

### Predictors of congenital heart defects

Poisson regression analysis, adjusted for all the possible risk factors, indicated that twin and multi-fetal infants were more likely to suffer from CHD than singleton infants (PRR:3.1, 95% CI:1.6, 6.1; Tab. 3). CHD was more prevalent in the children of mothers aged over 30 years than those under 25 years of age (PRR:1.6, 95% CI:1.1, 2.5). The prevalence rate ratio of CHD among mothers with more than 3 parity were much higher than that in mothers with only one parity (PRR:2.2, 95% CI:1.2, 4.2). Furthermore, family history of CHD could increase the risk of CHD among participants (PRR:9.8, 95% CI:5.3, 18.1), while the number of ultrasound examinations mothers underwent was not associated with rate of CHD.

**Table 3:** Predictors of any CHD among infants alive in Shaanxi province using a poisson regression

Predictors^c^	Univariate analysis^a^	Multivariate analysis^b^
	PRR (95% CI)	PRR (95% CI)
Infant Gender		
Male	Reference	Reference
Female	0.9 (0.7, 1.1)	0.9 (0.7, 1.2)
Fetal number		
Singleton	Reference	Reference
Twin and Multi-fetal	4.3 (2.4, 7.9)*	3.1 (1.6, 6.1)*
Mother's education		
College and above	Reference	Reference
Senior high school	0.9 (0.6, 1.3)	0.8 (0.5, 1.3)
Junior high school	0.9 (0.7, 1.3)	0.7 (0.5, 1.2)
Primary school	0.8 (0.5, 1.3)	0.6 (0.3, 1.1)
No education	1.3 (0.6, 3.0)	1.1 (0.4, 2.8)
Mother's age, year		
<25	Reference	Reference
25-29	1.2 (0.9, 1.8)	1.2 (0.8, 1.8)
≥30	1.6 (1.1, 2.4)*	1.6 (1.1, 2.5)*
Parity*		
1	Reference	Reference
2	1.1 (0.9, 1.5)	1.1 (0.8, 1.6)
≥3	2.6 (1.6, 4.3)*	2.2 (1.2, 4.2)*
Wealth index		
Poor	Reference	Reference
Medium	0.9 (0.6, 1.2)	0.9 (0.6, 1.3)
Rich	0.8 (0.6, 1.1)	0.9 (0.6, 1.3)
Family history of CHD*		
No	Reference	Reference
Yes	10.9 (6.2, 19.1)*	9.8 (5.3, 18.1)*
Residence during the pregnancy		
Permanent	Reference	Reference
Floating	0.6 (0.4, 0.8)*	0.6 (0.4, 0.9)*
Household registration		
Urban	Reference	Reference
Rural	0.9 (0.7, 1.3)	0.9 (0.6, 1.4)
Areas*		
Southern area	Reference	Reference
Central area	0.7 (0.5, 0.9)*	0.5 (0.4, 0.7)*
Northern area	0.5 (0.3, 0.7)*	0.4 (0.3, 0.6)*
Number of ultrasound examination	1.1 (0.9, 1.3)	1.1 (0.6, 1.4)

^a^Only one variable is entered into the poisson regression model

^b^All possible risk factors are entered into the poisson regression model

^c^Prevalence Rate Ratio (PRR) and 95% confidence interval are given to indicate the magnitude of change

*P < 0.05

The rate of CHD was lower in the floating population (PRR: 0.6, 95% CI: 0.4, 0.9) than permanent population, and in north (PRR: 0.4, 95% CI:0.3, 0.6) and central Shaanxi province (PRR: 0.5, 95% CI: 0.4, 0.7) than in south Shaanxi province. However, urban-rural difference and gender gap in CHD rate among the participants had not been found in surveyed areas.

## Discussion

The large population-based cross-sectional study conducted in Shaanxi province, northwest China, investigated the prevalence and epidemiological characteristics of CHD among infants born live between 2010 and 2013. We found that the prevalence of CHD was approximately 76.0 per 10000 live infants, and major defects may be a serious problem in the study. Furthermore, rate of CHD differed significantly with baseline characteristics among the participants in the surveyed areas.

In our study, 221 of 29098 participant infants were diagnosed with CHD. In 2007 a large population-based study in Beijing of fetuses and neonates screened for CHD by echocardiography, reported an incidence rate of 81.6 per 10000 births [15]. In four areas surrounding Shanghai, a previous surveillance program identified 75 cases of CHD among the 4006 fetuses and neonates who were scanned prenatally or neonatally, corresponding to an overall observed prevalence of 187.2 (95% CI: 14.8, 23.5) per 10000 births [7]. A population-based survey in Inner Mongolia reported a CHD prevalence of around 17.1 per 10000 births in 2008 [16]. A total of 1005052 births reported to the Birth Defects Monitoring Network of Guangdong province indicated an overall CHD prevalence of 52.4 per 10000 births (95% CI: 51.0, 53.8) [17]. Cross sectional studies indicated that the prevalence of CHD varied substantially between populations, from 82.8 per 10000 in Belgium to 12.2 per 10000 in Colombia [18, 19]. The prevalence of CHD in our study (76.0 per 10000 live births) is higher than that recently reported in Inner Mongolia (17.1 per 10000), Guangdong (52.4 per 10000), Colombia (12.2 per 10000), and similar to the rates reported in Beijing (81.6 per 10000) and Belgium (82.8 per 10000), but lower than that in Shanghai (187.2 per 1000 births). In our study population, we may have underestimated the rate of CHD as early death was not recorded, and minor CHD may have gone undetected. Therefore, we speculated that the prevalence of CHD in Shaanxi province may be much higher than 76.0 per 10000 live births.

In this study the prevalence of major and minor CHD were 26.1 and 49.8 per 10000 live infants, respectively. The most prevalent major categories of CHD were large atrial septal defect (ASD), large ventricular septal defect (VSD) and atrioventricular septal defect (AVSD). One study of CHD in areas near Shanghai, Eastern China reported a prevalence of 30.0 and 157.3 per 10000 births, respectively, for major and minor defects. The proportion of VSD (62.7%) was the highest among major defects in areas surrounding Shanghai, followed by ASD (18.7%) and ToF (5.3%) [7]. In Norway, research revealed a prevalence of 33.3 and 113.0 per 10000 fetuses for major and minor defects, respectively, and AVSD was the dominant category of major heart defects [5]. In our survey, only infants medically indicated for CHD underwent further diagnostic tests, so minor asymptomatic defects may have gone undetected. Furthermore, we used hospital records or congenital defects registers to identify cases of CHD. Due to asymptomatic or undetected conditions, however, many minor defects were likely overlooked, and nor reported in hospital records or congenital defects registers. Consequently, the percentage of major defects in our study was similar to that in other studies, while the proportion of minor defects was lower than in other studies.

In this study CHD prevalence varied between maternal age groups, and the risk for CHD in surveyed areas increased with maternal age after controlling for other risk factors using Poisson regression. Our results were consistent with previous reports [20–22]. Miller et al. reported that infants born to mothers older than 35 years were more likely to suffer from CHD than those born to younger mothers [21], and Reefhuis and Honein [22] also observed a positive association between maternal age and heart defects. In this study, higher maternal parity was also associated with CHD. A previous study conducted in Texas also revealed a slightly larger rate of birth defects among infants born to mothers with three or more prior births than those born to first-time mothers between 1999 and 2003 [23]. Simultaneously, twin and multi-fetal infants were positively associated with the increased risk of congenital heart disease. It was commonly considered that an excessive number of parturitions may lead to reproductive organ damage and dysfunction and increase the risk of CHD [24]. Further studies will be required to corroborate these findings and, if confirmed, to elucidate possible reasons for these associations.

Previous studies indicate that the relative risk of birth defects was increased in women with a history of similar defects in first-degree relatives [25]. For women with CHD, the prevalence of CHD among their offspring is approximately 10 times higher than that reported in the general population [26, 27]. Similarly, family history of CHD was also identified to an important predictor in our study. Many studies have suggested that sufficient maternal healthcare utilization, such as prenatal care and postnatal care, can efficiently reduce pregnancy-related diseases and newborn morbidity and mortality [28, 29]. Higher numbers of ultrasound examinations during pregnancy could indicate more accurate and early CHD diagnosis, which may have contributed to termination for significant CHD, however in this study we did not find rate of CHD to be associated with number of ultrasound examination. Thus, the role of periceonceptional ultrasound examination in CHD diagnosis should continue to be investigated in the surveyed areas.

In this study we also did not observe a significant difference between the rate of CHD in rural and urban areas. However, we found a significantly lower rate of CHD in floating populations than permanent populations, potentially due to differences in lifestyles and behaviors between these populations in Shaanxi province. The floating population were less likely to experience sustained environmental pollution and occupational exposure than the permanent population in the surveyed areas. However, the root causes of these differences remain unknown.

We also found that CHD incidence varied geographically in the surveyed population. The prevalence of CHD in north and central Shaanxi was lower than in south Shaanxi. Regional differences in CHD prevalence may be due to variations both in incidence and in mortality. In our study, parents in south Shaanxi were more likely to have access to antenatal scans, and hence the number of ultrasound examination was higher in south Shaanxi than in other areas. These interventions likely improved the rate of CHD detection in south Shaanxi, increasing the prevalence. Meanwhile, the discrepancy in rates of early termination of pregnancy between these areas may also have contributed to regional differences in rates of CHD. Another possible explanation is variations in the composition of the population in these study regions. In our study, south Shaanxi province has a larger proportion of floating and rural population than the other two areas, and, as previously noted, the prevalence of CHD was higher in the floating population. In a neighboring province, Inner Mongolia, the risk of birth defects was found to be higher in families living in rural areas than in urban areas [16]. Hence, region discrepancies in CHD could exist in Shaanxi province with a higher prevalence in south Shaanxi. These factors could explain in part the regional discrepancies in CHD prevalence across Shaanxi province. However, further studies will be required to clarify the contributions of these factors.

Here we report a large population-based cross-sectional survey conducted in Shaanxi province, in 30 counties or districts using a multi-stage sampling method, which reliably represents the recent prevalence of CHD in Shaanxi province. However, conclusions are limited by the scope of the study. Firstly, as a cross-sectional survey, only prevalence can be determined, and no conclusions can be drawn on the causes of CHD in this population. Secondly, all cases of CHD could not be included due to the limitations of our surveyed methods. For example, defects causing only undetectable symptoms are difficult to identify in early childhood; Hence, we suspect that our data underestimates the prevalence and burden of CHD. Nevertheless, the current study is the largest survey in Northwestern China, providing the most up-to-date data on CHD, and filling a gap in the knowledge of this geographical region.

## Conclusions

In conclusion, CHD among live infants is a serious health problem in Shaanxi province, Northwestern China, particularly in south Shaanxi. Twin and multi-fetal infants, family history among first-degree relatives and higher maternal age and parity were all positively correlated with CHD prevalence among live infants in the study areas. In addition, risk for CHD was lower in the floating population than in the permanent population. These findings may have an important public health policy implications for CHD intervention in northwest China.

## Additional file

Additional file 1: Questionnaire.doc Birth defects questionnaire: The questionnaire was used to collect the information on congenital heart disease and other related factors in the study. (DOC 240 kb)

## Abbreviations

AVSD: Atrioventricular septal defect
CHD: Congenital heart defects
CoA: Coarctation of the aorta
HLHS: Hypoplastic left heart syndrome
PRR: Prevalence rate ratio
Tetralogy of Fallot (ToF): Pulmonary valve stenosis (PS), Analysis of variance (ANOVA).
VSD: Ventricular septal defects
