# Supplementary material for: Prevalence and risk factors of congenital heart defects among live births: a population-based cross-sectional survey in Shaanxi province, Northwestern China
Source: BMC Pediatr. 2017 Jan 13;17:18. doi: 10.1186/s12887-017-0784-1 (PMC5237335; doi:10.1186/s12887-017-0784-1)
Supplement: Additional file 1: — Questionnaire.doc Birth defects questionnaire: The questionnaire was used to collect the information on congenital heart disease and other related factors in the study. (DOC 240 kb) [file 12887_2017_784_MOESM1_ESM.doc]

The survey of birth defects in Shaanxi Province

| **County** |  | **Township** | |  | **Village** | |  |
| --- | --- | --- | --- | --- | --- | --- | --- |
| **County Number (CC)** |  | **Township Number (TC)** | |  | **Village Number (VC)** | |  |
| **Participants Number(PC)** |  | | **Contact Telephone** | | |  | |
| **The eligibility of women participated in the survey:**  1. Since 2010, have you ever been pregnant? ①Yes ②No  2. Now, are you pregnant? ①Yes ②No ③Unknown  Can the women be included in the survey? ①Yes ②No  If choose “Yes”, please continue the survey. | | | | | | | |

| ***If the outcome of last pregnancy is live birth, and the child still survives，record name, gender, birthday, age (if they are twins, please record the name and gender of another child)*** | | | |
| --- | --- | --- | --- |
| **Child Name (CN)**  **Second child name (CN2)** |  | **Child gender(CG)**  **Second child gender(CG2)** | Male [1] Female [2]  Male [1] Female [2] |
| **Child birthday (CB)** | 20 ＿/＿ ＿/＿ ＿ | **Child age (CA)** | ____months |
| **Mother Name (MN)** |  | **Mother birthday (MB)** | 19＿＿/＿ ＿/＿ |
| **Father Name (FN)** |  | **Father birthday (FB)** | 19＿＿/＿ ＿/＿ |
| **Investiagtor Name**  **(IVN)** |  | **Survey date** | 201＿/＿ ＿/＿ ＿ |

Birth defects questionnaire-1

**(Fetuses and infants born after 2010**)

| A1-1  A1-2  A1-3  A1-4  A1-5  A1-6  A1-7  A1-8 | **Outcome of the last pregnancy**  ①live birth ② stillborn foetus, stillbirth ③ odinopoeia  ④ abactio ⑤ medical abortion ⑥ [spontaneous](javascript:void(0);) [abortion](javascript:void(0);) ⑦other______  **Fetal number of the last pregnancy** ① singleton ② twin ③ multi-fetal  **Fetal gender of the last pregnancy**  ①Male ②Female ③Unknown  **Reasons for abactio/ medical abortion/ odinopoeia** ① over-reproduction ② stillbirth ③birth defects ④ delay having a child ⑤other______  **If live born, the birth date of infant**  / /  gestational weeks ________weeks  **If odinopoeia/abortion, the date of odinopoeia/abortion** / / /  the weeks of  gestation termination: ________weeks | weeks  weeks |
| --- | --- | --- |
| A2-1A  A2-1B  A2-1C  A2-1D  A2-1E  A2-1F  A2-1G  A2-2  A2-3  A2-4  A2-5A  A2-5B  A2-6  A2-7 | **If child is still live, require a trained doctor to continue following questions and make cardiac auscultation**  Usually blue (cyanotic) ①Yes ②No ③Unknown  Often blue (cyanotic) when child cries with respiratory infections ①Yes ②No ③Unknown  Usually poor appetite ①Yes ②No ③Unknown  Repeatedly respiratory infections  ①Yes ②No ③Unknown  Clearly stunting ①Yes ②No ③Unknown  Pathological murmur in the anterior region of the heart ①Yes ②No ③Unknown  Family history of CHD among first-degree relatives ①Yes ②No ③Unknown  **During the the last pregnancy, whether there are confirmed cases of birth defects (including abortion, odinopoeia、stillborn foetus, stillbirth, live birth)**   1. Yes ②No ③Unknown   **If you select “Yes”, please give the types of birth defects (You may choose more than one options):**  01 Congenital heart disease(specific type )  02 [anencephaly](javascript:void(0);) 03 spina bifida  04 [encephalocele](javascript:void(0);) 05 [congenital](javascript:void(0);) [hydrocephalus](javascript:void(0);) 06 [cleft](javascript:void(0);) [palate](javascript:void(0);) 07 [harelip](javascript:void(0);) 08 cleft lip with palate 09 [microtia](javascript:void(0);)(include anotia)  13 [hypospadia](javascript:void(0);) 14 bladder exstrophy 10 other defects of [auricle](javascript:void(0);)(exclude [microtia](javascript:void(0);) and anotia)  11 [esophageal](javascript:void(0);) [atresia](javascript:void(0);) and stenosis 12 anorectum [atresia](javascript:void(0);) and stenosis(include aproctia )  15 [talipes](javascript:void(0);) [equinovarus](javascript:void(0);)(left or right) 16 [polydactyly](javascript:void(0);)(left or right) 17 [syndactylia](javascript:void(0);)(left or right)  18 limb [cripetura](javascript:void(0);)[include ectodactyle, ectrodactylia, cleft hand and foot]  Upper limb(left or right) Lower limbs(left or right)  19 congenital diaphragmatic hernia 20 [acromphalus](javascript:void(0);) 21 [gastroschisis](javascript:void(0);) 22  conjoined twins 23 [Down's](javascript:void(0);) [syndrome](javascript:void(0);)（ trisomy 21）  24 [metabolic](javascript:void(0);) [disease](javascript:void(0);)([phenylketonuria](javascript:void(0);), [albinsim](javascript:void(0);), G6PD deficiency and so on)  (specific type )  25 others (specific type )  [**Diagnostic**](javascript:void(0);) [**basis**](javascript:void(0);)**：** ①Obstetrician and Gynaecologist in hospitals and maternal and child health hospital above the county level  ②B ultrasound ③other______  **The time of confirmed diagnosis：** / /  Live births：age of confirmed diagnosis________months  Fetus: gestational weeks of confirmed diagnosis________weeks  [**Detail**](javascript:void(0);)**e**d [**description**](javascript:void(0);) **of birth defects:**  **If child with birth defects is still live, please take a photograph and encode:** | months  weeks |
| A3-1  A3-2  A3-3  A3-4  A3-4A  A3-4B | **Whether echocardiography and electrocardiography should be conducted for Child with suspected CHD in the first affiliated hospital of Xi’an Jiaotong University Health Science Center:**  ①Yes ②No  Mothers contacts information:  **Results of echocardiography and electrocardiography:**   1. normal 2. congenital heart disease(specific type ) 3. others(specific type ) |  |
| A4-1  A4-2  A4-3 | **The detection of birth defects questionnaire (**the supervisors are responsible for the work)   1. in line with medical records from hospital 2. can not find any medical records 3. other（ ）   **Encoding and clinical types of birth defects**  Clinical type: ① simple ②complex ③syndrome  Encoding: |  |

Birth defects questionnaire-2

**(If the infants are twins, please investigate another infant**)

| A1-1  A1-2  A1-3  A1-4  A1-5  A1-6  A1-7  A1-8 | **Outcome of the last pregnancy**  ①live birth ② stillborn foetus, stillbirth ③ odinopoeia  ④ abactio ⑤ medical abortion ⑥ [spontaneous](javascript:void(0);) [abortion](javascript:void(0);) ⑦other______  **Fetal number of the last pregnancy** ① singleton ② twin ③ multi-fetal  **Fetal gender of the last pregnancy**  ①Male ②Female ③Unknown  **Reasons for abactio/ medical abortion/ odinopoeia** ① over-reproduction ② stillbirth ③birth defects ④ delay having a child ⑤other______  **If live born, the birth date of infant**  / /  gestational weeks ________weeks  **If odinopoeia/abortion, the date of odinopoeia/abortion** / / /  the weeks of  gestation termination: ________weeks | weeks  weeks |
| --- | --- | --- |
| A2-1A  A2-1B  A2-1C  A2-1D  A2-1E  A2-1F  A2-1G  A2-2  A2-3  A2-4  A2-5A  A2-5B  A2-6  A2-7 | **If child is live, require a trained doctor to continue following questions and make cardiac auscultation**  Usually blue (cyanotic) ①Yes ②No ③Unknown  Often blue (cyanotic) when child cries with respiratory infections ①Yes ②No ③Unknown  Usually poor appetite ①Yes ②No ③Unknown  Repeatedly respiratory infections  ①Yes ②No ③Unknown  Clearly stunting ①Yes ②No ③Unknown  Pathological murmur in the anterior region of the heart ①Yes ②No ③Unknown  Family history of CHD among first-degree relatives ①Yes ②No ③Unknown  **During the the last pregnancy, whether there are confirmed cases of birth defects (including abortion, odinopoeia、stillborn foetus, stillbirth, live birth)**   1. Yes ②No ③Unknown   **If you select “Yes”, please give the types of birth defects (You may choose more than one options):**  01 Congenital heart disease(specific type )  02 [anencephaly](javascript:void(0);) 03 spina bifida  04 [encephalocele](javascript:void(0);) 05 [congenital](javascript:void(0);) [hydrocephalus](javascript:void(0);) 06 [cleft](javascript:void(0);) [palate](javascript:void(0);) 07 [harelip](javascript:void(0);) 08 cleft lip with palate 09 [microtia](javascript:void(0);)(include anotia)  13 [hypospadia](javascript:void(0);) 14 bladder exstrophy 10 other defects of [auricle](javascript:void(0);)(exclude [microtia](javascript:void(0);) and anotia)  11 [esophageal](javascript:void(0);) [atresia](javascript:void(0);) and stenosis 12 anorectum [atresia](javascript:void(0);) and stenosis(include aproctia )  15 [talipes](javascript:void(0);) [equinovarus](javascript:void(0);)(left or right) 16 [polydactyly](javascript:void(0);)(left or right) 17 [syndactylia](javascript:void(0);)(left or right)  18 limb [cripetura](javascript:void(0);)[include ectodactyle, ectrodactylia, cleft hand and foot]  Upper limb(left or right) Lower limbs(left or right)  19 congenital diaphragmatic hernia 20 [acromphalus](javascript:void(0);) 21 [gastroschisis](javascript:void(0);) 22  conjoined twins 23 [Down's](javascript:void(0);) [syndrome](javascript:void(0);)（ trisomy 21）  24 [metabolic](javascript:void(0);) [disease](javascript:void(0);)([phenylketonuria](javascript:void(0);), [albinsim](javascript:void(0);), G6PD deficiency and so on)  (specific type )  25 others (specific type )  [**Diagnostic**](javascript:void(0);) [**basis**](javascript:void(0);)**：** ①Obstetrician and Gynaecologist in hospitals and maternal and child health hospital above the county level  ②B ultrasound ③other______  **The time of confirmed diagnosis：** / /  Live births：age of confirmed diagnosis________months  Fetus: gestational weeks of confirmed diagnosis________weeks  [**Detail**](javascript:void(0);)**e**d [**description**](javascript:void(0);) **of birth defects:**  **If child with birth defects is still live, please take a photograph and encode:** | months  weeks |
| A3-1  A3-2  A3-3  A3-4  A3-4A  A3-4B | **Whether echocardiography and electrocardiography should be conducted for Child with suspected CHD in the first affiliated hospital of Xi’an Jiaotong University Health Science Center:**  ①Yes ②No  Mothers contacts information:  **Results of echocardiography and electrocardiography:**   1. normal 2. congenital heart disease(specific type )   ③others(specific type ) |  |
| A4-1  A4-2  A4-3 | **The detection of birth defects questionnaire (**the supervisors are responsible for the work)   1. in line with medical records from hospital 2. can not find any records 3. other（ ）   **Encoding and clinical types of birth defects**  Clinical type: ① simple ②complex ③syndrome  Encoding: |  |

Family questionnaire

| E2 | Mother nationality E2：①Han ②Hui ③Other______ | E2______ |
| --- | --- | --- |
| E3 | Mother education E3：①College and above ②Senior high school ③Junior high school  ④Primary school ⑤No education | E3______ |
| E4 | Mother marriage E4：①first marriage ②remarriage ③divorce ④widow ⑤others_____ | E4______ |
| E5 | Household registration E5：①Urban ②Rural | E5______ |
| E6 | How many people are there in your family? E6a______  In which，number of children E6b______  Number of boys E6c ______ | E6a_____  E6b_____  E6c_____ |
| E7  E8 | **If you are urban residents，please reponse**  Household income per month E7a______  Household expenditure per month E7b______  Housing E7c ①private housing(housing size m2) ②rental housing  Automobile E7d ①Yes（total price ______Yuan） ②None  **If you are rural residents，please reponse**  [Agriculture](javascript:void(0);) income per year E8a______Yuan  Part-time work income per year E8b______Yuan  Other income per year E8c______Yuan  Total expenditure per year E8d______Yuan  Housing E8e：①apartment ②brick houses ③adobe houses  Household appliances（television, refrigerator, washing machine, air conditioner, computer）E8f： ①Yes (how many?____) ②No  Automobile（may choose more than one）E8g： ①family car ② agricultural vehicle  ③motorcycle, electric bikes ④None | E7a_____  E7b_____  E7c_____  E7d_____  E8a_____  E8b_____  E8c_____  E8d_____  E8e_____  E8f_____  E8g_____ |
| E9 | GPS location of surveyed area(village or community)：  Longitude E9a______  Latitude E9b______  Altitude E9c______meters  The area is your residence during the last pregnancy? E9d：Yes ②No  If “No”, Please give permanent residence during the last pregnancy: ______Province______county(district)______township(street)______village(communitiy) | E9a_____  E9b_____  E9c_____  E9d_____ |
| E10 | What is the frequency of alcohol intake during pregnancy? E10a  ①per day ②3-4times/week ③1-2 times/week ④ <1/week ⑤never | E10a_____ |
| E11 | Have you ever smoked during pregnancy? E11  ①never  ②more than 1 cigerate per week, and less than 3 months of duration  ③more than 1 cigerate per day, and 3-6 months of duration  ④more than 5 cigerate per day, and more than 6 months of duration  ⑤unknown | E11______ |
| E12 | During pregnancy, are there smokers in your family? E12a  ①Yes, not avoid ②Yes, avoid ③None  During pregnancy, what is your frequency of passively inhaling smoke for > 15 minutes per day? E12b  ①nearly everyday ②>3 days/week ③1-3 days/week ④<1 day/week ⑤unknown | E12a_____  E12b_____ |
| E13 | What is the frequency of tea consumption during pregnancy? E13a  ①No ②At times, cups/week E22b ③Everyday, cups/day E22c | E13a_____  E13b_____  E13c_____ |
| E14 | What is the frequency of coffee consumption during pregnancy? 14a  ①No ②At times, cups/week E23b ③Everyday, cups/day E23c | E14a_____  E14b_____  E14c_____ |
| E15 | During pregnancy, did you accept ultrasound examination? E15a ①Yes times ②No  Where is your ultrasound examination during pregnancy? E15e：  ①Hospital above county level  ②Maternal and child care service centre above county level  ③Township hospital  ④Village clinic  ⑤Private clinic ⑥Others | E15a_____  E15b_____  E15c_____  E15d_____  E15e_____ |

| Reproductive history (RH)-1 | | | | | | |
| --- | --- | --- | --- | --- | --- | --- |
|  | | | | | |  |
| **Pregnancy sequence** | **The first pregnancy** | **The second pregnancy** | **The third pregnancy** | **The fourth pregnancy** | **The fifth pregnancy** | **The sixth pregnancy** |
| **Termination date of pregnancy** | / / | / / | / / | / / | / / | / / |
| **Gestational weeks of pregnancy ternimation** |  |  |  |  |  |  |
| **Pregnancy Outcome: （please fill in the numbers in the below blanks）**  1. spontaneous abortion； 2. abactio； 3. medical abortion； 4. odinopoeia； 5. stillborn foetus, stillbirth； 6. live birth(eutocia, accouche)； 7. live birth(cesarean delivery)； 8. ectopic gestation； 9. vesicular mole | | | | | | |
|  |  |  |  |  |  |  |
| The gender of fetuses and infants | 1.male 2.female 3.Unknown | 1.male 2.female 3.Unknown | 1.male 2.female 3.Unknown | 1.male 2.female 3.Unknown | 1.male 2.female 3.Unknown | 1.male 2.female 3.Unknown |
| Whether the infant survive | 1.Yes 2.No | 1.Yes 2.No | 1.Yes 2.No | 1.Yes 2.No | 1.Yes 2.No | 1.Yes 2.No |
| Birth weight of infants(grams) | 1._____grams 2.Unknown | 1._____grams 2.Unknown | 1._____grams 2.Unknown | 1._____grams 2.Unknown | 1._____grams 2.Unknown | 1._____grams 2.Unknown |
| Are there birth defects among infants | 1.male 2.female 3.Unknown | 1.male 2.female 3.Unknown | 1.male 2.female 3.Unknown | 1.male 2.female 3.Unknown | 1.male 2.female 3.Unknown | 1.male 2.female 3.Unknown |
| If “Yes”, specific types |  |  |  |  |  |  |
| Whether the infants is still live | 1.live 2.dead | 1.live 2.dead | 1.live 2.dead | 1.live 2.dead | 1.live 2.dead | 1.live 2.dead |
| If dead, what is the reason? |  |  |  |  |  |  |
| If dead, please give the age of death |  |  |  |  |  |  |
| The summary based on above data: [gravidity](javascript:void(0);) parity spontaneous abortion abactio medical abortion odinopoeia:total male female  eutocia：total male female preterm：total male female birth defects：total male female stillborn foetus and stillbirth：total male female newborn death：total male female infant death：total male female | | | | | | |

Family history (FH)—paternal line

|  | Grandfather | Grandmother | Father | Brothers and sisters of father |  | Grandfather | Grandmother | Father | Brothers and sisters of father |
| --- | --- | --- | --- | --- | --- | --- | --- | --- | --- |
| Heart disease | 1congenital heart disease  2rheumatic heart disease  3coronary disease  4 others  5 No 6Unknown | 1congenital heart disease  2rheumatic heart disease  3coronary disease  4 others  5 No 6Unknown | 1congenital heart disease  2rheumatic heart disease  3coronary disease  4 others  5 No 6Unknown | 1congenital heart disease  2rheumatic heart disease  3coronary disease  4 others  5 No 6Unknown | Tuberculosis | 1Yes  2No  3Unknown | 1Yes  2No  3Unknown | 1Yes  2No  3Unknown | 1Yes  2No  3Unknown |
| [Renal](javascript:void(0);) [disease](javascript:void(0);) | 1Yes 2No 3Unknown | 1Yes 2No 3Unknown | 1Yes 2No 3Unknown | 1Yes 2No 3Unknown | Blind | 1Yes 2No 3Unknown | 1Yes 2No 3Unknown | 1Yes 2No 3Unknown | 1Yes 2No 3Unknown |
| Chronic liver disease | 1Yes 2No 3Unknown | 1Yes 2No 3Unknown | 1Yes 2No 3Unknown | 1Yes 2No 3Unknown | Deaf | 1Yes 2No 3Unknown | 1Yes 2No 3Unknown | 1Yes 2No 3Unknown | 1Yes 2No 3Unknown |
| Hypertension | 1Yes 2No 3Unknown | 1Yes 2No 3Unknown | 1Yes 2No 3Unknown | 1Yes 2No 3Unknown | Dumb | 1Yes 2No 3Unknown | 1Yes 2No 3Unknown | 1Yes 2No 3Unknown | 1Yes 2No 3Unknown |
| Anemia | 1Yes 2No 3Unknown | 1Yes 2No 3Unknown | 1Yes 2No 3Unknown | 1Yes 2No 3Unknown | Amentia | 1Yes 2No 3Unknown | 1Yes 2No 3Unknown | 1Yes 2No 3Unknown | 1Yes 2No 3Unknown |
| Hyperthyreosis | 1Yes 2No 3Unknown | 1Yes 2No 3Unknown | 1Yes 2No 3Unknown | 1Yes 2No 3Unknown | Diabetes | 1Yes 2No 3Unknown | 1Yes 2No 3Unknown | 1Yes 2No 3Unknown | 1Yes 2No 3Unknown |
| Hypothyroidism | 1Yes 2No 3Unknown | 1Yes 2No 3Unknown | 1Yes 2No 3Unknown | 1Yes 2No 3Unknown | Hemophilia | 1Yes 2No 3Unknown | 1Yes 2No 3Unknown | 1Yes 2No 3Unknown | 1Yes 2No 3Unknown |
| Mental disease | 1Yes 2No 3Unknown | 1Yes 2No 3Unknown | 1Yes 2No 3Unknown | 1Yes 2No 3Unknown | Asthma | 1Yes 2No 3Unknown | 1Yes 2No 3Unknown | 1Yes 2No 3Unknown | 1Yes 2No 3Unknown |
| Epilepsy | 1Yes 2No 3Unknown | 1Yes 2No 3Unknown | 1Yes 2No 3Unknown | 1Yes 2No 3Unknown | Genital system | 1Yes 2No 3Unknown | 1Yes 2No 3Unknown | 1Yes 2No 3Unknown | 1Yes 2No 3Unknown |
| Kaschin-Beck disease | 1Yes 2No 3Unknown | 1Yes 2No 3Unknown | 1Yes 2No 3Unknown | 1Yes 2No 3Unknown | sexually transmitted disease | 1Yes 2No 3Unknown | 1Yes 2No 3Unknown | 1Yes 2No 3Unknown | 1Yes 2No 3Unknown |
| Cancer | 1Yes 2No 3Unknown | 1Yes 2No 3Unknown | 1Yes 2No 3Unknown | 1Yes 2No 3Unknown | Birth defects | 1Yes 2No 3Unknown | 1Yes 2No 3Unknown | 1Yes 2No 3Unknown | 1Yes 2No 3Unknown |
| Specific cancer |  |  |  |  | Specific type |  |  |  |  |
| Surgery | 1Yes 2No 3Unknown | 1Yes 2No 3Unknown | 1Yes 2No 3Unknown | 1Yes 2No 3Unknown | Others | 1Yes 2No 3Unknown | 1Yes 2No 3Unknown | 1Yes 2No 3Unknown | 1Yes 2No 3Unknown |
| Specific tye |  |  |  |  | Specific type |  |  |  |  |
| Grandparents (including cousins) consanguinity 1 Yes 2 No 3 Unknown | | | | | | | | | |

Family history (MH)—Maternal line

|  | Grandfather | Grandmother | Mother | Brothers and sisters of father |  | Grandfather | Grandmother | Mother | Brothers and sisters of father |
| --- | --- | --- | --- | --- | --- | --- | --- | --- | --- |
| Heart disease | 1congenital heart disease  2rheumatic heart disease  3coronary disease  4 others  5 No 6Unknown | 1congenital heart disease  2rheumatic heart disease  3coronary disease  4 others  5 No 6Unknown | 1congenital heart disease  2rheumatic heart disease  3coronary disease  4 others  5 No 6Unknown | 1congenital heart disease  2rheumatic heart disease  3coronary disease  4 others  5 No 6Unknown | Tuberculosis | 1Yes  2No  3Unknown | 1Yes  2No  3Unknown | 1Yes  2No  3Unknown | 1Yes  2No  3Unknown |
| [Renal](javascript:void(0);) [disease](javascript:void(0);) | 1Yes 2No 3Unknown | 1Yes 2No 3Unknown | 1Yes 2No 3Unknown | 1Yes 2No 3Unknown | Blind | 1Yes 2No 3Unknown | 1Yes 2No 3Unknown | 1Yes 2No 3Unknown | 1Yes 2No 3Unknown |
| Chronic liver disease | 1Yes 2No 3Unknown | 1Yes 2No 3Unknown | 1Yes 2No 3Unknown | 1Yes 2No 3Unknown | Deaf | 1Yes 2No 3Unknown | 1Yes 2No 3Unknown | 1Yes 2No 3Unknown | 1Yes 2No 3Unknown |
| Hypertension | 1Yes 2No 3Unknown | 1Yes 2No 3Unknown | 1Yes 2No 3Unknown | 1Yes 2No 3Unknown | Dumb | 1Yes 2No 3Unknown | 1Yes 2No 3Unknown | 1Yes 2No 3Unknown | 1Yes 2No 3Unknown |
| Anemia | 1Yes 2No 3Unknown | 1Yes 2No 3Unknown | 1Yes 2No 3Unknown | 1Yes 2No 3Unknown | Amentia | 1Yes 2No 3Unknown | 1Yes 2No 3Unknown | 1Yes 2No 3Unknown | 1Yes 2No 3Unknown |
| Hyperthyreosis | 1Yes 2No 3Unknown | 1Yes 2No 3Unknown | 1Yes 2No 3Unknown | 1Yes 2No 3Unknown | Diabetes | 1Yes 2No 3Unknown | 1Yes 2No 3Unknown | 1Yes 2No 3Unknown | 1Yes 2No 3Unknown |
| Hypothyroidism | 1Yes 2No 3Unknown | 1Yes 2No 3Unknown | 1Yes 2No 3Unknown | 1Yes 2No 3Unknown | Hemophilia | 1Yes 2No 3Unknown | 1Yes 2No 3Unknown | 1Yes 2No 3Unknown | 1Yes 2No 3Unknown |
| Mental disease | 1Yes 2No 3Unknown | 1Yes 2No 3Unknown | 1Yes 2No 3Unknown | 1Yes 2No 3Unknown | Asthma | 1Yes 2No 3Unknown | 1Yes 2No 3Unknown | 1Yes 2No 3Unknown | 1Yes 2No 3Unknown |
| Epilepsy | 1Yes 2No 3Unknown | 1Yes 2No 3Unknown | 1Yes 2No 3Unknown | 1Yes 2No 3Unknown | Genital system | 1Yes 2No 3Unknown | 1Yes 2No 3Unknown | 1Yes 2No 3Unknown | 1Yes 2No 3Unknown |
| Kaschin-Beck disease | 1Yes 2No 3Unknown | 1Yes 2No 3Unknown | 1Yes 2No 3Unknown | 1Yes 2No 3Unknown | sexually transmitted disease | 1Yes 2No 3Unknown | 1Yes 2No 3Unknown | 1Yes 2No 3Unknown | 1Yes 2No 3Unknown |
| Cancer | 1Yes 2No 3Unknown | 1Yes 2No 3Unknown | 1Yes 2No 3Unknown | 1Yes 2No 3Unknown | Birth defects | 1Yes 2No 3Unknown | 1Yes 2No 3Unknown | 1Yes 2No 3Unknown | 1Yes 2No 3Unknown |
| Specific cancer |  |  |  |  | Specific type |  |  |  |  |
| Surgery |  |  |  |  | Others | 1Yes 2No 3Unknown | 1Yes 2No 3Unknown | 1Yes 2No 3Unknown | 1Yes 2No 3Unknown |
| The uterus and accessories |  | 1Yes 2No 3Unknown | 1Yes 2No 3Unknown | 1Yes 2No 3Unknown | Specific type |  |  |  |  |
| Other | 1Yes 2No 3Unknown | 1Yes 2No 3Unknown | 1Yes 2No 3Unknown | 1Yes 2No 3Unknown |  |  |  |  |  |
| Specific type |  |  |  |  |  |  |  |  |  |
| Are there any blood relationship between you and your husband (including the relationship between cousins)? 1 Yes 2 No 3 Unknown  Grandparents (including cousins) consanguinity 1 Yes 2 No 3 Unknown | | | | | | | | | |
